# Modeling health impact of global health programs implemented by Population Services International

**DOI:** 10.1186/1471-2458-13-S2-S3

**Published:** 2013-06-17

**Authors:** Hongmei Yang, Susan Duvall, Amy Ratcliffe, David Jeffries, Warren Stevens

**Affiliations:** 1 Population Services International, Washington DC, USA; 2 Independent global health consultant, Seattle, WA, USA; 3 Independent consultant, Fajara, The Gambia; 4 Independent health economics consultant, Boston, MA, USA

## Abstract

**Background:**

Global health implementing organizations benefit most from health impact estimation models that isolate the individual effects of distributed products and services - a feature not typically found in intervention impact models, but which allow comparisons across interventions and intervention settings. Population Services International (PSI), a social marketing organization, has developed a set of impact models covering seven health program areas, which translate product/service distribution data into impact estimates. Each model's primary output is the number of disability-adjusted life-years (DALYs) averted by an intervention within a specific country and population context. This paper aims to describe the structure and inputs for two types of DALYs averted models, considering the benefits and limitations of this methodology.

**Methods:**

PSI employs two modeling approaches for estimating health impact: a macro approach for most interventions and a micro approach for HIV, tuberculosis (TB), and behavior change communication (BCC) interventions. Within each intervention country context, the macro approach determines the coverage that one product/service unit provides a population in person-years, whereas the micro approach estimates an individual's risk of infection with and without the product/service unit. The models use these estimations to generate per unit DALYs averted coefficients for each intervention. When multiplied by program output data, these coefficients predict the total number of DALYs averted by an intervention in a country.

**Results:**

Model outputs are presented by country for two examples: Water Chlorination DALYs Averted Model, a macro model, and the HIV Condom DALYs Averted Model for heterosexual transmission, a micro model. Health impact estimates measured in DALYs averted for PSI interventions on a global level are also presented.

**Conclusions:**

The DALYs averted models offer implementing organizations practical measurement solutions for understanding an intervention's contribution to improving health. These models calculate health impact estimates that reflect the scale and diversity of program operations and intervention settings, and that enable comparisons across health areas and countries. Challenges remain in accounting for intervention synergies, attributing impact to a single organization, and sourcing and updating model inputs. Nevertheless, these models demonstrate how DALYs averted can be viably used by the global health community as a metric for predicting intervention impact using standard program output data.

## Background

Estimating the impact of health promotion programs is essential in the global health sphere, and a growing priority among donors [1]. Donor funding from key players in international health, such as The World Bank, The Global Fund to Fight AIDS, Tuberculosis and Malaria, and the United States Agency for International Development (USAID), has become increasingly based on performance [2-4]. To demonstrate performance and maintain stakeholder interest in its program initiatives, global health implementing organizations need to be able to articulate the impact of health promotion programs and use evidence to improve decision making.

Mathematical models have been widely used in global health to predict disease burden and estimate the differential impact of a variety of interventions. To model disease burden estimates, The Global Burden of Disease (GBD) study provides a standard methodology [5]. Standard models do not exist for assessing intervention impact, however. As modelers can utilize different approaches to generate impact estimates and this type of modeling can serve diverse purposes, individual organizations and researchers have developed many different models for policy decisions and program planning. For example, models have been used to inform the scale up of male circumcision initiatives, to guide decision making and the allocation of resources for family planning services, and to plan malaria control initiatives [6-8].

Until recently, models for estimating intervention impact did not fully address the unique measurement needs of implementing organizations which utilize product marketing and distribution techniques, as well as behavior change communication, to encourage healthy behaviors. These organizations were challenged when predicting health impact estimates due to one or more of the following reasons:

1) Intervention impact models did not directly estimate the health impact of an individual unit of product or health service. While the Lives Saved Tool (LiST) is widely used for health impact estimations, it requires coverage as an input at the population level (i.e., percent of a population using a given a product) [9], not the number of products distributed or services provided - a standard output measure regularly tracked by implementing organizations. Therefore, these organizations need models that can convert these data into estimates of per-unit impact in order to quantify the effect of the distributed products and services. Such quantification can help an organization demonstrate its specific contribution to population-level health outcomes, an important distinction given the variety of players who promote the same health interventions in many countries, such as government agencies, social marketing organizations, and commercial sector organizations.

2) With the increase in integrated programs, intervention impact models often estimated the impact of a set of interventions, such as integrated maternal and neonatal health programs, instead of isolating the effects of each individual intervention [10,11]. However, implementing organizations want the flexibility to scope, predict, and track the impact of their specific interventions, which can be aggregated or disaggregated. Therefore, models are needed to produce outputs that compare the potential or predicted impact of each individual intervention to enable informed decision making within the specific country or program context.

3) Intervention impact models did not cover the breadth of products and services often offered by implementing organizations with broad program portfolios. Instead, the models focused on a single health area [6] or a narrow age range [9]. This limited scope prohibited comparisons across different interventions for planning or tracking. As implementing organizations are increasingly expanding their scope of work, they are, therefore, interested in estimating impact consistently across health areas and age ranges with impact measures that allow comparisons.

For these reasons, Population Services International, a non-profit social marketing organization implementing programs in 65 developing countries around the globe, began developing its own set of models for estimating health intervention impact based on distribution and service data in 2007. As PSI tracks its product distribution and service delivery in a wide range of health areas from malaria and HIV to maternal health and child survival, it needed a set of models that would isolate the effect of each individual intervention used in all of its health program areas in each country where PSI works. It also needed to understand the estimated impact of a single product or service unit, its primary type of output data. On their own, distribution and service data could not be used to make viable cross-program and cross-country impact comparisons because product/service outputs did not account for the context where the intervention occurred, i.e., epidemiological differences.

Therefore, to make viable impact estimates and comparisons, PSI chose to adopt the disability-adjusted life year, the standard unit developed in the early 1990s by the World Health Organization's Global Burden of Disease study to estimate burden of disease (BOD) in a population, as its primary impact measure. The DALY is defined as the number of healthy years of life lost to premature death or disability as a result of a disease condition. It measures the gap between current health status and an ideal situation in which everyone lives into old age free of disease and disability. As the DALY accounts for both morbidity and mortality, and allows comparisons across health areas, interventions, geographic areas, and age groups [12], PSI adopted it for estimating program impact. PSI models the number of DALYs that is averted, not lost, by each of its interventions within a specific country context. These models are called DALYs averted models.

Currently, PSI has a DALYs averted model for nearly all of its products and services covering its seven major health program areas: family planning (FP), HIV/AIDS and other STIs, tuberculosis, malaria, maternal health (e.g., abortion, clean delivery practice, and micronutrient deficiencies), child health (e.g., acute respiratory infection, diarrhea, micronutrient deficiencies, and malnutrition), and cervical cancer. In total, there are 27 discrete models for 44 interventions, each delivering a different product or service. Some of the models estimate health impact across multiple health conditions, such as basic care packages for people living with HIV and AIDS (covering malaria, diarrhea, HIV, and FP), male circumcision among neonates and adults (covering HIV, HSV-2, and HPV), and clean needles and syringes for injection drug users (IDUs) (covering HIV, HBV, and HCV). Some models estimate health impact of interventions specific to high-risk populations or sub-groups, such as STI treatment kits and naloxone for treating overdose among IDUs. Finally, five models assess the impact of behavior change activities promoting both PSI-branded and non-PSI prevention and treatment products as well as behavior that does not involve products (e.g., reducing number of sexual partners). See Additional file 1 for a list of PSI's models.

The DALYs averted models are not used for health impact evaluation, that is, to assess PSI's programs by answering cause-and-effect questions. Rather, these models are used to translate program outputs - usually tracked by the number of products and services sold or distributed - into estimates of health impact. PSI measures program output and models DALYs averted. The modeled results allow for impact comparisons across its diverse program and geographic areas, informing PSI decisions, guiding priority setting, motivating staff, and demonstrating performance to stakeholders.

In this paper, we describe the methodology of PSI's DALYs averted models, explaining how product and service distribution data are modeled into health impact estimates, with the final model output being the number of DALYs averted by a PSI intervention. We describe the structure and inputs for two different types of DALYs averted models used at PSI, as well as demonstrate the results of each one. As these models may be of interest to other implementing organizations that engage in global health social marketing, we also discuss the limitations of our models and share lessons learned during the modeling process.

## Methods

### Overview of PSI's DALYs averted models

Each DALYs averted model is designed to estimate the health impact of one unit of a product or one service delivered (e.g., one condom, one intrauterine device (IUD) insertion, or one pre-packaged treatment for malaria) that PSI offers. The modeling output is a set of coefficients that represent the number of DALYs averted by a single unit or service corresponding to each country where PSI works. These coefficients are multiplied by the sales, distribution, or service provision data (referred to forthwith as "distribution data") for the intervention in the country of interest, to convert the reported distribution data into an estimate of the total number of DALYs averted by the intervention within that country. Effectively, this calculation gives an estimate of the DALYs averted that can be attributed to the products distributed or services provided by PSI in a country.

We model most interventions separately to account for differences in the nature of the disease, data availability, and complexity of health impact components. At the highest level, PSI's DALYs averted models can be classified as being either macro, modeled at the population level, or micro, modeled at the individual user or client level.

We take a macro approach for the vast majority of PSI's models, those that estimate the impact of interventions in health areas other than HIV/AIDS and TB (i.e., malaria, FP, maternal and child health). These models use population-level epidemic data (e.g., incidence rate and death rate) to estimate burden of disease or the risks of infection and death, and assume that interventions reduce morbidity and mortality to estimate the number of DALYs averted. Many published mathematic models estimating health impact, including LiST, have taken this approach [9].

For the health area of HIV/AIDS and TB, we use a micro approach because people's behavior plays an important role in the transmission of these two diseases. Thus, these models start by estimating an individual's risk of infection based on risk behaviors and the epidemic situation in the country of interest. Unlike PSI's macro DALYs averted models, probabilistic transmission models are required for these estimations. Natural history or disease progression is also considered to estimate risk of death after being infected. Finally, we also take a micro approach to model the impact of BCC interventions because PSI's current BCC interventions concern health conditions (e.g., HIV/AIDS, heroin overdose death) that rely on probabilistic models to estimate an individual's risk of developing the condition, not population-level burden of disease estimates.

PSI's interventions that cross multiple disease areas, such as the basic care package for people living with HIV/AIDS (PLHIV), require more complex modeling to estimate DALYs averted. These models combine multiple PSI DALYs averted models, sometimes combining both approaches for interventions that involve HIV/AIDS. This paper does not discuss these combined models, although the structure, inputs, outputs, and operation can be inferred from the description of the two basic types.

### Structure of PSI's health impact estimation models

#### Theoretical basis: incremental impact

PSI's DALYs averted models are based on the principle of incremental impact, an estimate of the difference in disease burden between a baseline scenario when an intervention is absent and a follow-up scenario when an intervention is present. The change that occurs in the presence of the intervention is the incremental impact. In theory, the disease burden in the follow-up scenario should be lower than baseline levels because PSI's intervention aims to reduce the disease burden. It is important to understand that the models are theoretical, informed by best evidence but without any actual measurement of disease reduction. Because PSI's interventions involve product distribution and service provision in many cases, to estimate incremental impact in the DALYs averted models, we estimate the number of years that a user is protected (or person-years of protection) by the product or service throughout the product/service's lifespan.

Substitution will affect these estimates of incremental impact. Substitution is best understood as the proportion of PSI-branded products and services distributed that may substitute for products and services from either the public sector or other organizations. The substitution rate ranges from zero (no substitution at all) to 100% (complete substitution). The higher the substitution rate, the smaller the incremental impact. Across PSI's interventions, BCC models have considered substitution in modeling through pre- and post-intervention behavioral data. Substitution is not an issue for certain services that target first-time users or users who need replacement of an intervention product, such as male circumcision and IUD insertion, respectively. Similarly, substitution does not need to be considered where treatment or testing is warranted by an incident case, like treatment for malaria or HIV voluntary counseling and testing. For certain preventive products, we take substitution into account by including market share in the model. Substitution, therefore, only affects a certain set of PSI interventions, primarily preventive products with short-term effectiveness, such as safe water solution and long-lasting, insecticide-treated bednets (LLINs). Where PSI distributes these products through social marketing, there are insufficient data to take substitution into account. Therefore, we assume zero substitution in our modeling for preventive products other than condoms. As a result, the impacts estimated by these models do not necessarily represent the net impact of PSI's programs, but only the gross impact of the products distributed.

#### Model parameters and data inputs

In general, the DALYs averted model parameters follow those typically used in academic mathematical models of health interventions: 1) measures of disease burden; 2) estimates of intervention efficacy or effectiveness; and 3) the change in coverage or use over the modeled time period [9]. PSI's models uniquely consider intervention coverage based on the per-unit product/service. They model the impact of product/services distributed, not the impact based on population changes in coverage. Therefore, in PSI's models, coverage is based on the coverage that one unit provides in person-years to a user.

PSI's macro models use these three inputs. The key parameters required in PSI's macro models are Burden, Efficacy/Effectiveness, Attrition, Utilization, and Time frame, or BEAUTy. As risk of infection replaces burden in the HIV and TB DALYs averted models, the key parameters for these micro models are Risk of Infection, Efficacy/Effectiveness, Attrition, Utilization, and Time frame, or REAUTy.

Burden is based on the epidemiology of a health condition or disease in the country of interest, measured by incidence and mortality rates. We rely on The Global Burden of Disease publications as an important source for estimates of mortality. Morbidity is more difficult to find. In cases where published literature or nationally representative surveys are not available, we use regional estimates. Table 1 shows a list of data sources for each model parameter.

**Table 1:** Data sources of key DALYs averted model parameters

Parameter	Data Source
Burden (macro models)	*Mortality Data:* ■ WHO Global Burden of Disease reports [[18,26] ■ Demographic and Health Surveys (DHS)[27] ■ Multiple Indicator Cluster Surveys [28] *Morbidity Data:* ■ DHS ■ Published literature

Risk of Infection (micro models)	■ Published literature ■ UNAIDS or WHO reports (for epidemic data) [29,30] ■ PSI population-based surveys (for behaviors such as sexual behavior or drug use behavior) [31]

Efficacy/Effectiveness	■ Published randomized controlled trials, community randomized trials ■ Child Health Epidemiology Reference Group (CHERG) reviews [32] ■ Cochrane Literature Review [33] ■ WHO Global Burden of Disease reports

Attrition, or Wastage	■ PSI supply chain studies (as of 2011) ■ PSI population-based surveys (as of 2011) ■ Communication with PSI programmers Note: Wastage data are rarely available in the published literature so wastage levels were assumed until 2011.

Utilization (including adherence)	*Utilization Data:* ■ PSI population-based surveys ■ USAID CYP conversion factor for family planning products [34] ■ Published literature *Adherence Data:* ■ Published literature ■ PSI product usage studies (e.g., pill counts in HAART program) ■ Communication with experts in the field Note that protective efficacy/effectiveness studies, such as CHERG reviews, are likely to have adherence/compliance data embedded in them already.

Time frame	Defined as one year. For products/services with lifespans longer than one year, all years of impact are counted in the year of distribution.

Disability Weight	Global Burden of Disease and Risk Factors [18]

Duration of Disease or Disability	Published literature

Age at Death due to Disease or Disability	■ Age distribution data of age at death ■ Weighted estimates of age at death based on age group data from Global Burden of Disease and Risk Factors [18]

Life Expectancy	WHO, 2011 (for Japanese life expectancy data) [35]

For the health area of HIV, we express the risk of infection as a function of an individual's behavior (e.g., condom use, male circumcision (MC) status, or needle sharing among IDUs), the current epidemic of HIV, and, in the case of sexual transmission, the MC rate and STI rate. New infections progress over time during which individuals may receive antiretroviral treatment when they are eligible for treatment, and therefore, experience better survival.

For the health area of TB, we also take a micro approach as with HIV, but we need to use a more complex, compartmentalized model because of the nature of the disease. With TB, individuals progress through four stages: susceptible (uninfected); exposed (infected, but not yet infectious); infectious; and recovered, in which the risk of re-infection exists. Therefore, we apply risk of infection to the susceptible stage, risk of progression to the exposed stage, risk of transmission to the infectious stage and risk of re-infection to the recovered stage. While this model is stratified by HIV status, we do not account for movement from HIV-negative to HIV-positive status within the current model.

Efficacy/effectiveness indicates how well a treatment or a public health intervention achieves a therapeutic or protective effect. Efficacy measures the capacity under ideal circumstances, such as clinical trials or laboratory settings, while effectiveness measures the capacity under real-life conditions. Ideally, all health impact models would be based on effectiveness because it measures the effect closer to users' context and usage experience. In our modeling, data availability and quality determine whether efficacy or effectiveness is used. All of the interventions listed in Additional file 1 use effectiveness estimates in modeling except for: antibiotics to treat STIs (i.e., STI Kit); male and female condom; male circumcision; safe abortion; and cervical cancer screening and treatment. Models for these interventions use estimates of efficacy.

Attrition, or wastage, refers to loss of product at different levels of the distribution system. Such attrition is particularly important to consider when assessing program impact for organizations that distribute products because product distribution often targets providers or retailers rather than clients or users. Wasted products are those that were procured but never used for their intended purpose, an idea that is not new [13,14]. Possible sources of product wastage include being expired before reaching the consumer, being used for other purposes, and being destroyed. Reasons why this attrition occurs include: 1) consumer loss or non-use, particularly for those products that are not used frequently and last a long time, such as naloxone; and 2) supply chain inefficiencies, which may cause products to not be delivered [13,14]. Accounting for product attrition helps set PSI's models apart from other approaches that model program impact, helping PSI avoid over-claiming health impact from its programs.

Utilization refers to the use of products/services by the individuals who are the intended intervention targets. For many products and services, especially non-single dose products such as artemisinin-based combination therapy (ACT) for malaria treatment, utilization combines data on use as well as adherence, or compliance. Adherence refers to the proportion of products/services that is actually used by the purchaser, allowing for some purchasers who may not use the product as directed, including not using the full amount in the case of products with dosages. As adherence levels less than 100% reduce product usage rates, adherence is an important factor in assessing the potential impact of an intervention. However, a key challenge when factoring adherence into health impact estimates is the wide variance in adherence reported by intervention studies. Even in clinical trials where patients were well selected, reported average adherence rates ranged from 43-78% among patients receiving treatment for chronic conditions [15]. These varying rates of compliance can help explain the disparity in results between the same interventions under different circumstances. Similarly, this variance may also help explain the wide range of 'effect' identified in many protective efficacy/effectiveness reviews that commonly include compliance data [16].

The time frame set for all of the models is one year. PSI estimates health impact on a yearly basis because burden of disease indicators are measured in a year (e.g., morbidity and mortality rates). For products or services whose effective lifespan is greater than one year, PSI claims all years of impact in the year that the product or service was provided. For example, since male circumcision is assumed to last 20 years, we sum up the impact of one MC service on preventing HIV and other sexually transmitted diseases over the course of 20 years, and report it in the year the client received a circumcision surgery.

We use an additional set of parameters to calculate the DALYs averted: disability weight, duration of condition, age at death from condition, and average life expectancy. We follow the methodology of calculating DALYs according to the Disease Control Priorities Project [17,18]. While a 3% time discount rate applies to future years, age weighting is not applied (i.e., DALY(0.03, 0)).

The models are implemented deterministically. This deterministic implementation does not account for uncertainty in the point estimates used for the model parameters. However, depending on the parameters, uncertainty can be incorporated in one of two ways: 1) by developing empirical distributional assumptions about the parameter, or 2) by using range data or confidence intervals typically obtained from a meta-analysis. The models allow users to run sensitivity analyses to address the uncertainty issue.

#### Model outputs

PSI's health impact estimation models produce estimates of impact of one unit of a product or service on the health of a population. Model outputs include: cases, or new infections, averted, deaths averted, and DALYs averted per product or service. For products and services in family planning, the models also estimate couple-years of protection (CYP), pregnancies averted, and maternal deaths averted. In addition, family planning models produce estimates of the number of child deaths averted as a result of the longer birth spacing intervals afforded by contraception. All of these estimates can be produced at a national level, or by subgroup, focusing on specific age groups and/or geographic areas (e.g., rural and urban).

While all of the outputs are important and useful health impact measures, the key metric that is modeled is DALYs averted, and in particular, the DALYs averted coefficients that are multiplied by a country's distribution data to yield estimates of the total impact of a program. As the other measures listed above (e.g., cases averted) do not allow for comparisons of impact across health areas, DALYs averted is the primary model output.

When modeling these outputs, modelers modify the calculations and assumptions slightly for those interventions targeting or having impacts on multiple diseases. In these DALYs averted models, the models assume that new cases averted are the sum of all possible cases because individuals can have multiple diseases at the same time. However, as an individual can die only once, even though she or he may have multiple infections at the time of death, cause of death is considered when calculating deaths averted. The individual's death will be counted only toward the disease that is the recorded cause of death. Interaction between diseases is also considered if evidence exists, such as TB and HIV as well as HIV and malaria.

### Examples of how PSI's DALYs averted models operate

To illustrate how the DALYS averted models work, this section describes the specific inputs, structure, and model operation of two of PSI's DALYs averted models. The Water Chlorination Model is an example of a model that takes a macro approach, while the HIV Condom Model is an example of one that uses a micro approach. Even though the inputs will change, all of PSI's models will follow the structure and operation of one of these examples.

#### Structure and operation of Water Chlorination DALYs Averted Model (macro approach)

PSI promotes household water treatment products because they are one of the most effective and cost-effective means of preventing waterborne disease in development and emergency settings. These products include a safe water solution, *PUR*, and *Aquatabs*, all of which are point-of-use treatments that use chlorination to treat drinking water at a microbiological level. The initial purpose of PSI's household water treatment programs is to reduce diarrhea cases and deaths in children under five. Because the products are used at the household level, all residents benefit from using the products. Therefore, PSI's household water treatment programs target populations of all ages but mainly focus on children. This example estimates the impact of point-of-use water treatment products on the likelihood of diarrhea episodes and death among children under five years of age. Tables 2 and 3 list the specific data sources and values used for each parameter in this model, shown as country-specific parameters and fixed parameters.

**Table 2:** Country-specific parameters and data sources in the Water Chlorination Model for children under five

Parameter	Input	Source
Diarrhea morbidity rate in children under five, by country	Varies by country	DHS

Diarrhea mortality rate in children under five, by country	Varies by country	DHS

Demographic data (population size, proportion of children under five)	Varies by country	UNPD [36]

**Table 3:** Fixed parameters and data sources in the Water Chlorination Model for children under five

Parameter	Input	Source
Protective effectiveness of chlorinated, point-of-use water treatment products	47%	Clasen et al., 2006 [21]

Wastage rate of household water treatment products	15%	Communication with PSI water treatment technical experts

Liters of water treated per unit of product:		Product usage guidelines
• Safe water solution (various brand names)	1000	
*• PUR*	10	
*• Aquatabs*	20	

Liters of water per person per day for drinking and cooking in households with unpiped water	3	Tumwine et al., 2002 [19]; Thompson et al., 2003 [20]

Duration of diarrhea episodes (years)	0.03	Lopez et al., 2006 [17]

Disability weight of diarrhea episodes	0.105	Mathers et al., 2006 [18]

Age at death from diarrheal disease for children under five (years)	1.8	Calculated based on IMR, U5MR and proportion of under-five deaths due to diarrhea from: WHO, 2007 [37]; Black et al., 2010 [38]

Life expectancy (years)	83.1	WHO, 2011 [35]

In this model, a macro approach is taken. As the water treatment intervention is distributed to households, the model begins by calculating the number of household-years of protection from one unit of the PSI water treatment product within the entire population of interest. We then isolate the age group that we want to measure program impact for. In the example, we show this for children under five.

First, we use the following equation to translate one unit of water treatment product to household-years of protection (HYP) (i.e., years of effective protection of all household members from diarrhea):

(1) $$\begin{array}{l} \text{Household-years of protection}=[\text{(Number of liters treated per unit of product) /(household}\\ \text{size * per capita usage per day * 365)}]\text{ * (protective effectiveness) * (}1-\text{wastage)} \end{array}$$

The first part of the equation determines the utilization. We ascertained per capita usage per day from studies showing the number of liters of water per day that a household without piped water uses on average for drinking and cooking [19,20]. Household size varies from country to country. The multiplier, 365, is included to account for the time frame of one year.

We obtained the protective effectiveness associated with household water treatment using chlorination from the most recent Cochrane Review [21], an examination of 30 randomized and quasi-randomized controlled trials of various drinking water interventions in diarrhea-endemic settings. The review's meta-analysis confirmed that household-level, point-of-use interventions through chlorination are effective in preventing diarrhea in children and adults. In addition, the findings suggest that compliance is already embedded in the effectiveness rates, and this compliance is positively associated with protective effectiveness. Utilization equations used in other PSI macro models may need to include a variable for the compliance rate.

We then modify household-years of protection by attrition, with the remainder (1-wastage) used to calculate a more accurate estimate of product distribution in the population.

Second, the number of children under five years of age who are protected per unit of product is estimated by using the following equation:

(2) $$\begin{array}{c} \text{Number of children under five protected per unit of product}=\text{HYP * household }\\ \text{size * proportion of children under five in a household} \end{array}$$

We use general demographic data for household size and the proportion of children under five in a household because there is no evidence indicating that households that treat their water (or use PSI products) are demographically different from those that do not.

Third, to estimate the burden averted from the PSI water treatment intervention, the model uses the number of children under five protected per unit of product to produce estimates of cases averted and deaths averted per unit of product. This estimation is done by multiplying the number of children under five who benefited from treated water in the country of interest during the specified year by the country's diarrhea morbidity and mortality rates of children under five:

(3) $$\begin{array}{c} \text{Diarrhea cases averted among children under five per unit of product}=\text{number }\\ \text{of children under five protected per unit of product * diarrhea morbidity in children under five} \end{array}$$

(4) $$\begin{array}{c} \text{Diarrhea deaths averted among children under five per unit of product}=\\ \text{number of children under five protected per unit of product * diarrhea mortality in children under five} \end{array}$$

Finally, to estimate DALYs averted, we use the following equation:

(5) $$\begin{array}{c} \text{DALYs averted among children under five per unit of product}=\text{(cases averted}\\ \text{among children under five per unit of product * duration of diarrhea * disability weight of diarrhea)}\\ +\text{ [deaths averted among children under five per unit of product * (life expectancy}-\text{age at death}\\ \text{from diarrhea) }\left(\text{which is discounted at 3\% for future years}\right)\text{]} \end{array}$$

#### Structure and operation of HIV Condom DALYs Averted Model for heterosexual transmission (micro approach)

PSI employs condom social marketing programs to target sexually active adults and youth, widely distributing male condoms through traditional outlets (e.g., pharmacies, clinics, grocery stores, etc.) and/or specific venues where sexual partners are more likely to meet (e.g., hotels, night clubs, vending machines in red light districts, etc.). PSI offers condoms at varying price points to reach individuals at different income levels, including free of charge for those who cannot pay. This example of the HIV Condom Model focuses on heterosexual transmission and male condoms, estimating the impact of male condoms on reducing HIV transmission between men and women. Like the macro model above, we assume the time period for this intervention is one year. Tables 4 and 5 list the specific data sources and values for the major country-specific and fixed parameters in this model, with Additional file 2 showing the adjustment calculations used for a few of the parameters.

**Table 4:** Country-specific parameters and data sources used in the HIV Condom Model

Parameter	Input	Source
Country-specific sexual behavior data:	Varies by country	PSI population-based TRaC surveys*[31]
• Number of sexual partners by relationship in past year		
• Number of sexual contacts with each type of partner in past year		
• Use of any brand of condom with each type of partner		
• Use of PSI condom with each type of partner		

Country-specific HIV prevalence among general adults	Varies by country	UNAIDS, 2010 [39]

Country-specific male circumcision rate	Varies by country	Williams et al., 2006 [40]

HIV prevalence among female commercial partners	Varies by country	Calculated**

STI prevalence among general adults	Varies by country	Calculated**

STI prevalence among female sex workers (FSWs)	Varies by country	UNAIDS, 2010 or calculated when unavailable**

*When country-specific sexual behavior data are unavailable from either published sources or PSI population-based surveys in the country of interest, we use combined data from three population-based surveys conducted by PSI in Angola, Zambia, and Zimbabwe among adults aged 15-49 years.

**When data are not readily available from published sources, we calculate HIV prevalence among commercial sex workers and STI prevalence in both the adult population and FSWs, adjusting data for FSW HIV prevalence from WHO/UNAIDS Epidemiological Fact Sheets on HIV/AIDS and STIs (2008), data for STI prevalence in general adult population from WHO (2001) and data for HIV prevalence in the general adult population by region from UNAIDS/WHO (2006). (Adjustment calculations shown in Additional file 2.) STI prevalence among commercial sex partners is set at 80% for PSI platforms lacking available data.

**Table 5:** Fixed parameters and data sources used in the HIV Condom Model

Parameter	Input	Source
Per-act infectivity of HIV when index partner has no symptom and both partners are negative for other STIs	0.0005	Gray et al., 2001 [24]; Boily et al., 2009 [41]

Per-act infectivity of HIV when index partner has acute infection of HIV and both partners are negative for other STIs	0.0047	Pilcher et al., 2004 [42]; Wawer et al., 2005 [43]

Effect of STI on HIV transmission	5	Satten et al., 1994 [25]; Rottingen et al., 2001 [44]

Protective efficacy of male condoms	90%	Pinkerton et al., 1997 [45]

Protective efficacy of male circumcision	60%	Auvert et al., 2005 [46]; Bailey et al., 2007 [47]; Gray et al., 2007 [48]

Acute period of HIV infection (days)	54	Pilcher et al., 2004 [42]

Duration of HIV and AIDS when left untreated (years)	10 for HIV; 2 for AIDS	Todd et al., 2007 [49]

Disability weight of HIV	0.135 for HIV; 0.505 for AIDS	Mathers et al., 2006 [18]

Age at infection of HIV (years)	26	Assumed based on the fact that most sero-conversions occur within the 25-29 year-old age group (Todd et al., 2007 [49])

Wastage of condoms	10%	Communication with PSI programmers

Life expectancy at birth (years)	83.1	WHO, 2011 [35]

First, we determine an individual's risk of infection by the human immunodeficiency virus from sexual contact with discordant partners during the course of one year. Various factors contribute to this risk, all of which must be modeled in different scenarios to determine the cumulative risk of HIV infection for the individual. Partner type is one risk factor, encompassing regular, casual, and commercial (female sex workers) partners. Another risk factor is the total number of sexual partners within the past year. To identify cut-off points of risk groups defined by the total number of sexual partners within the past year, we used data collected from PSI's population-based behavior surveys in three sub-Saharan African countries (Angola (unpublished PSI data), Zimbabwe [22], and Zambia [23]). The data indicated that the majority (77.3%) of sexually active people aged 15-49 (*n *= 5,297) had only one partner in the past year, 11% had two partners, 5% had 3-4 partners, 3.7% had 5-9 partners and 2.5% had at least 10 partners. Based on the distribution of the data, we identified five cut-off points to create five risk groups for defining individual behavior in terms of partner quantity:

• Only 1 partner in the past year

• 2 partners in the past year

• 3-4 partners in the past year

• 5-9 partners in the past year

• ≥ 10 partners in the past year

As additional risk factors, the model considers the HIV status of these partners as well as the number of sexual contacts with these partners (in which one contact is equivalent to one episode of sexual intercourse). Finally, the per-act infectivity of HIV transmission also determines an individual's risk of infection. This infection risk per act is influenced by a multitude of cofactors, including STI infection status within the partnership, baseline circumcision status of the male partner, infection stage of the HIV-positive partner and, importantly, the intervention behavior that this DALYs averted model focuses on: condom use during sexual intercourse.

**Figure 1:**
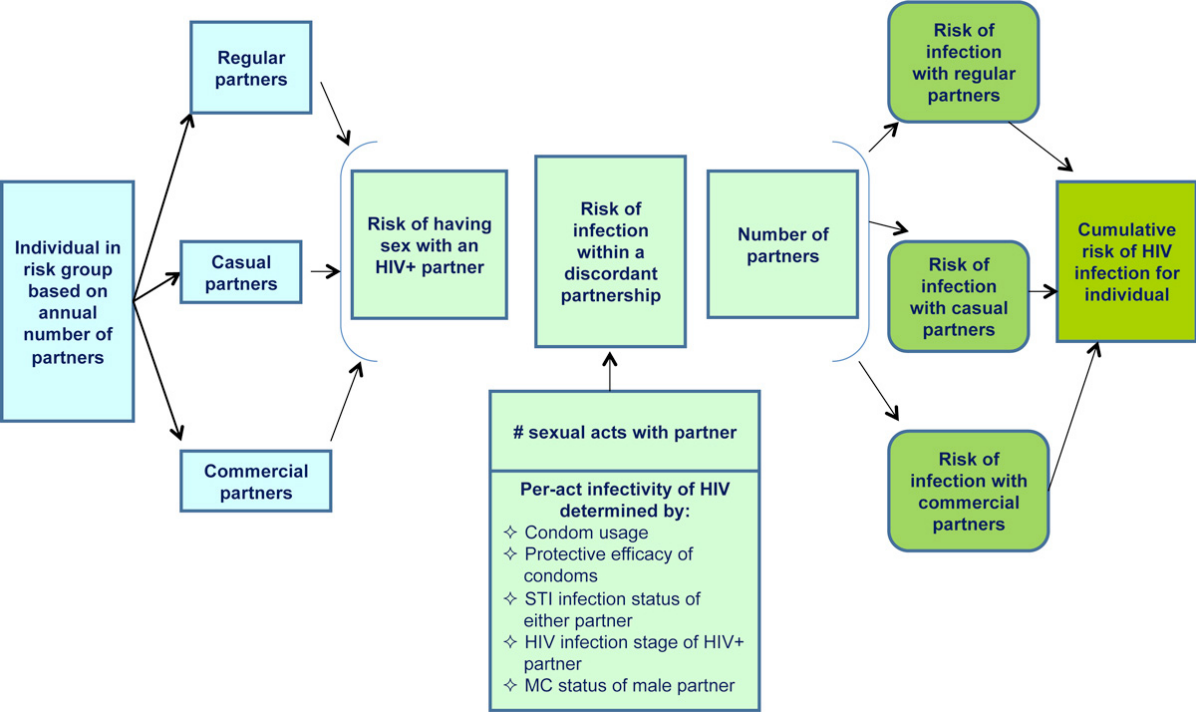
**Risk factors for HIV infection through heterosexual transmission, determined for each type of partner and each risk group**.

Figure 1 illustrates how all of these factors contribute to an individual's risk of HIV infection. For individuals in each of the five risk groups, the model runs a scenario in which the individual has *x *partners, *y *sexual contacts with each type of partner, and *z *sexual contacts protected by a condom. These scenarios also incorporate the other infection risk factors (e.g., STI infection status within partnership, male circumcision status, and HIV infection stage), leading to infection risk estimates for each type of partner. Taken together, these estimates create a cumulative risk of infection estimate for the individual in each risk group.

To calculate the risk of heterosexual infection for an individual in one of these risk groups, the HIV condom model uses a set of transmission risk equations based on Bernoulli probability theory [24,25]. This paper provides an overview of the model's operation.

The probability of an HIV-negative partner becoming infected in a heterosexual, discordant partnership during the modeled time period (one year) can be represented by Equation 6 below:

(6) $$P=1-{\left(1-p\times \lambda \right)}^{m}$$

In this equation,  p represents the prevalence of HIV in the general adult population. Therefore, a person who is HIV-negative has  p chances of having sexual intercourse with a partner who is HIV-positive, assuming partner choice is random (in terms of probability theory). The variable, *m *represents the number of sexual partners in a year. The final variable,  λ, is the probability of HIV transmission within a discordant partnership, represented by the equation, $1-{\left(1-\gamma \right)}^{n}$, resulting in the following (Equation 7) when substituted into  λ in Equation 6 above:

(7) $$P=1-(1-p\times {\left(1-{\left(1-\gamma \right)}^{n}\right)}^{m}$$

In Equation 7, *n *represents the number of sexual contacts with each partner. Therefore, the probability of infection through heterosexual contact is associated both with the number of sexual partners (*m) *and the number of sexual contacts with each partner (*n)*. This equation for  λ assumes the probability of HIV transmission during each sexual contact is independent.

In Equation 7, the variable,  γ, refers to the per-act infectivity of HIV during unprotected vaginal intercourse. Calculating this per-act infectivity is determined through various probabilistic combinations of the risk cofactors that an HIV-negative person may encounter in an HIV-positive partner (e.g., STI infection status in either partner, circumcision status of male partner). When condoms are used, we assume that the per-act infectivity is reduced by the protective efficacy of the condom (90%). These various equations are detailed in Additional file 3.

As Equation 7 does not take the type of sexual partner into account, it is modified further to consider the three main types of sexual partners: regular, casual, and commercial. Assuming these three categories are used, the modified equation for estimating the probability of infection through heterosexual contact (equation 8) is expressed as follows:

(8) $$P=1-\underset{\begin{array}{c} i=\mathrm{regular}\\ \mathrm{casual}\\ \mathrm{commercial} \end{array}}{\prod }\left(1-P_{i}\right)=1-\underset{\begin{array}{c} i=\mathrm{regular}\\ \mathrm{casual}\\ \mathrm{commercial} \end{array}}{\prod }\left(1-p_{i}*{\left(1-{\left(1-\gamma_{i}\right)}^{n_{i}}\right)}^{m_{i}}\right)$$

In this equation, $p_{i}$ refers to the HIV prevalence among different partner types. For regular and casual partners, it uses the HIV prevalence rate in the general adult population. For commercial partners, it uses the HIV prevalence rate among female sex workers in that country. Using this equation, the model determines the likelihood of HIV infection for an individual within each risk group.

Second, we determine the reduction in transmission risk by PSI's intervention. To achieve that, an individual's cumulative risk of infection at a follow-up level of condom use (post-intervention) is subtracted from the cumulative risk of infection at baseline (pre-intervention). Comparing baseline with follow-up, the only difference is the level of condom use.

Third, we estimate the impact of one PSI condom in averting new HIV infections through the following equation:

(9) $$\begin{array}{c} \text{Estimated number of new infections averted per condom per year}=\\ \text{(Reduction in HIV transmission risk per person per year / Utilization of PSI condoms per person }\\ \text{per year) * (1}-\text{wastage)} \end{array}$$

For calculating the number of PSI condoms used per person per year, utilization is estimated based on sexual activity and condom utilization data recorded by the PSI country office. This utilization estimate is adjusted by attrition (1 - wastage) to calculate a more accurate estimate of condom usage in the population.

Finally, the model estimates the DALYs averted coefficient for PSI's male condom used in interventions aimed at preventing heterosexual HIV transmission. For this disease that currently lacks a cure, the model assumes that all new infections will die of AIDS-related diseases. Therefore, we do not calculate deaths averted as we did for the Water Chlorination Model above; rather, we only use the number of new infections averted (equivalent of cases averted). The calculations above provide this number. To estimate the DALYs averted coefficient, the HIV Condom Model uses the standard equation from all PSI models:

(10) $$\begin{array}{c} \text{DALYs averted per unit of product}=\text{(new infections averted per unit of product * duration}\\ \text{of HIV * disability weight of HIV)}+\text{(new infections averted per unit of product * duration of AIDS *}\\ \text{disability weight of AIDS)}+\text{[new infections averted per unit of product * (life expectancy}-\text{age at}\\ \text{death from AIDS)]} \end{array}$$

For determining the duration of HIV and age at death from AIDS, the model assumes that individuals contract HIV at age 26. It also assumes that highly active antiretroviral therapy (HAART) was not widely available back in 2007 when the HIV Condom Model was developed. Therefore, the effect of antiretroviral therapy (ART) is not included in the model. Given the wide availability of ART in many developing countries in 2010s, this will significantly over-estimate the impact of condoms. We are in the process of improving the HIV Condom Model by including the effect of ART. In addition, the durations of HIV, AIDS, and years lost due to premature death are discounted at 3% for future years.

## Results

To demonstrate how the PSI DALYs averted models operate in practice, we modeled health impact estimates for each of the examples provided, based on data from countries where PSI operates. These estimates were the model outputs (i.e., coefficients for cases averted, deaths averted, and DALYs averted) for each of the models described above. Then, to show the total health impact of PSI's health interventions in 2012, we applied these model outputs to distribution data, yielding the estimated number of DALYs averted for that intervention product/service in each country where PSI operates.

For the Water Chlorination Model example, health impact estimates for one PSI water treatment product called *PUR *are presented in Table 6 for Cambodia and Cameroon, based on the assumed distribution of one million units of *PUR*. As shown, the potential impact of *PUR *is considerably greater in Cameroon than Cambodia. The distribution of one million units of *PUR *during one year in Cameroon is estimated to avert twice the number of diarrheal episodes in the population and nearly five times the number of deaths averted, in comparison to the estimated number of episodes and deaths averted in Cambodia. As a result, the overall annual health impact by the intervention in Cameroon is estimated to be 176 DALYs averted per one million units of *PUR*, more than triple the number estimated for Cambodia, 54.8 DALYs averted per one million units of *PUR*.

**Table 6:** Model outputs from Water Chlorination DALYs Averted Model for *PUR *intervention in Cambodia and Cameroon

Country	Product	All-age Episodes Averted per Unit	All-age Deaths Averted per Unit	All-age DALYs Averted per Unit (Model Coefficient)	Product Distribution Volume	All-age Episodes Averted	All-age Deaths Averted	All-age DALYs Averted
Cambodia	*PUR*	0.001723	1.61E-6	5.48E-5	1,000,000	1,723	1.61	54.8

Cameroon	*PUR*	0.003367	5.42E-6	1.76E-4	1,000,000	3,367	5.42	176

The impact of water purification using chlorine is larger in Cameroon than in Cambodia mainly due to different population-level epidemiologic and demographic data that are key model inputs. Compared with Cambodia, Cameroon has a higher burden of diarrheal disease for children under five. Cameroon's diarrhea morbidity rate is 5.34 episodes per child per year versus 3.12 episodes in Cambodia; in terms of the under-five diarrhea mortality rate, Cameroon has a rate of 0.009 deaths per child per year, much higher than Cambodia's rate of 0.00445 deaths per child. Moreover, the proportion of children under five in a household numbers much higher in Cameroon than Cambodia (15% vs. 9.5%).

The impact estimates per unit of *PUR *correspond proportionally to the total estimated number of DALYs averted for the year from PSI's diarrhea prevention interventions using *PUR*. Applying DALYs averted per unit (the model coefficient) to the actual product distribution volume during one year gives us the estimate of the health impact of the program in that year. Table 7 shows the total estimated impact of all of PSI's *PUR *programs worldwide in 2012.

**Table 7:** DALYs averted for *PUR *by PSI programs in 2012*, by country

Country	All-age DALYs Averted Coefficient for *PUR*	2012 *PUR *Distribution	All-age DALYs Averted by *PUR*, 2012
Congo-Kinshasa	0.000195	3,729,019	728

Dominican Republic	0.000032	758,640	24

Ethiopia	0.000150	5,665,462	847

Kenya	0.000120	7,374,447	885

Panama Warehouse**	0.000041	2,411,040	99

Malawi	0.000137	12,017,029	1,648

Namibia	0.000087	1,104,352	96

Nigeria	0.000169	9,360	2

Rwanda	0.000183	1,799,146	329

South Sudan	0.000118	1,457,588	171

Tanzania	0.000143	4,261,200	610

Uganda	0.000137	3,759,394	515

**Total**		**44,346,677**	**5,954**

*Only those countries implementing a PSI *PUR *intervention in 2012 are shown.

**Panama Warehouse serves Columbia, Dominican Republic, Haiti, and Panama

Table 8 summarizes the model outputs from the HIV Condom DALYs Averted Model for heterosexual transmission in two selected countries: Thailand and Zimbabwe. Since the number of new infections averted per unit of condom is quite small, we show condoms per infection averted. Within both countries, the number of condoms required to prevent one infection steadily decreases as the population's risk of HIV infection increases. The impact of PSI condom use is the smallest in the lowest risk group (characterized by having only one sexual partner during one year) and greatest in the highest risk group (characterized by having more than 10 sexual partners during one year).

**Table 8:** Model outputs from HIV Condom DALYs Averted Model intervention in Thailand and Zimbabwe

Country	Risk Group	Condoms per Infection Averted	Condoms per Death Averted	DALYs Averted per Unit (model coefficient)	Product Distribution Volume in One Year*	New Infections Averted in One Year*	Deaths Averted in One Year*	DALYs Averted in One Year*
Thailand	Only 1 partner	167,224	167,224	1.14E-4				

	2 partners	33,784	33,784	5.65E-4				

	3-4 partners	11,862	11,862	1.61E-3				

	5-9 partners	5,263	5,263	3.65E-3				

	≥10 partners	3,817	3,817	5.10E-3				

	All risk groups	8,000	8,000	2.43E-3	1,000,000	125	125	2,430

Zimbabwe	Only 1 partner	10,417	10,417	1.83E-3				

	2 partners	7,463	7,463	2.56E-3				

	3-4 partners	4,484	4,484	4.25E-3				

	5-9 partners	2,577	2,577	7.48E-3				

	≥10 partners	2,183	2,183	8.91E-3				

	All risk groups	3,690	3,690	5.24E-3	1,000,000	271	271	5,240

*Values for each individual risk group are not shown because product distribution data are only collected for all risk groups and cannot be disaggregated.

When the health impact estimates for each risk group from Thailand and Zimbabwe are compared with each other, notable differences can be seen. For all groups and all outputs, Zimbabwe's impact estimates per condom are more than double the size of Thailand's estimates. For the all-risk group that expresses the combined impact from all risk groups, the number of condoms needed to prevent one infection is 3,690 in Zimbabwe and 8,000 in Thailand. Accordingly, the estimated DALYs averted per unit is 5.24E-3 for all risk groups in Zimbabwe, compared with 2.43E-3 in Thailand. Based on these coefficients, if one million condoms are distributed by PSI in one year, these condoms are projected to avert 271 new infections and deaths in Zimbabwe, whereas, in Thailand, they will avert just 125 new infections and deaths. As a result, Zimbabwe's male condom intervention is expected to avert 5,240 DALYs if one million male condoms are distributed, versus 2,430 DALYs averted from the same intervention in Thailand.

To determine the actual estimate of DALYs averted in a year by PSI's condom interventions - the estimated health impact of male condoms in one year, we applied DALYs averted per unit (the model coefficient) to the actual volume of condom distribution during that year. Table 9 shows the estimated global impact of a sample of PSI's male condom programs in 2012, chosen to illustrate the variation in results across different geographies and differing program outputs. See Additional file 4 for the complete list of PSI countries engaged in male condom programs in 2012 and their estimated global impact.

**Table 9:** HIV DALYs averted for male condoms by selected PSI programs in 2012*, by country

Country	HIV DALYs Averted Coefficients for Male Condoms	Male Condom Distribution, 2012	HIV DALYs Averted by Male Condoms, 2012
Angola	0.002793	7,722,752	21,567

Benin	0.002315	9,474,758	21,930

Botswana	0.006008	3,222,809	19,362

Cambodia	0.000970	19,011,469	18,435

Cameroon	0.003683	21,427,386	78,923

China	0.000210	262,482	55

Costa Rica	0.000127	1,092,387	138

Cote d'Ivoire (+AIMAS**)	0.003391	22,174,988	75,194

Guatemala	0.000468	10,211,611	4,781

Haiti	0.001742	2,124,288	3,700

India	0.000510	221,303,250	112,900

Madagascar	0.001962	9,070,108	17,799

Mexico	0.000127	25,578	3

Myanmar	0.001158	21,993,848	25,475

Nicaragua	0.000078	5,159,547	402

Nigeria	0.003018	213,739,536	645,122

Pakistan	0.000132	103,110,124	13,589

Romania	0.000956	4,335,875	4,145

South Africa	0.004907	70,367,916	345,290

South Sudan	0.003243	1,288,580	4,179

Swaziland	0.006132	1,642,992	10,075

Tanzania	0.003862	68,724,288	265,423

**Total for all countries with interventions^**		**1,095,906,825**	**2,655,973**

*Countries were selected to show a range of results from the diverse geographical regions where PSI implemented a male condom intervention in 2012.

**AIMAS is l'Agence Ivorienne de Marketing Social, a local NGO in Côte d'Ivoire that PSI works closely with to implement male condom interventions.

^These totals do not reflect the distribution and DALYs averted for male condoms from the sample of countries presented here. Instead, it shows the totals for all PSI countries with male condom interventions in 2012. See additional file 4 for the complete list of results from all countries.

The estimated health impact of all of PSI's interventions in 2012, in terms of DALYs averted, are presented in Additional file 5, arranged in decreasing order of the health impact of each intervention. In 2012, PSI's long-lasting insecticide-treated nets, male condoms, and pre-packaged ACT malaria treatment kits averted the largest number of DALYs.

## Discussion

PSI's DALYs averted models enable direct translation of product and service distribution data into estimates of health impact, converting one unit of a product or service into a quantifiable approximation of that unit's reduction in the disease burden - the number of DALYs averted. In doing so, these models provide global health implementing organizations with a powerful tool for understanding an intervention's contribution to improving health, at an organization-wide, country office, or program level. The model coefficients easily facilitate the calculation of health impact estimates from monthly or quarterly distribution data, allowing the dissemination of regular and timely impact reports. As such, an implementing organization can internally track the progress of its efforts in improving people's health over time, by country or region as well as by health area. In addition, the use of actual program output data is invaluable for demonstrating tangible and credible performance to donors and other stakeholders as well as for engaging in target setting and planning. In conjunction with burden of disease numbers, PSI's models provide planners, programmers, and policy makers with a tool to prioritize interventions and to examine the potential of expanding their health impact by taking different strategies. In conjunction with cost analysis, they allow programmers and planners a means for understanding the cost-effectiveness of their programs by producing cost per DALY of each product/service, thereby enabling better strategic thinking and greater efficiencies in global health program implementation.

The two health impact estimation model structures described in this paper - the micro models for HIV, TB, and current BCC interventions, and the macro model for other interventions - are designed for wide application as well as customization. Unlike most health impact mathematical models, PSI's set of DALYs averted models enables impact estimation for a wide variety of health program areas, from diarrhea to HIV, reflecting the diversity of health behaviors that social marketing organizations seek to encourage. These structures also facilitate customization for each intervention setting, enabling the application of proven intervention efficacy or effectiveness estimates to any risk or burden context where PSI works. As each country's specific population, epidemiologic, and product utilization data can be input into the model parameters, the DALYs averted models generate health impact estimates specific to that setting. Moreover, it is possible to break down study populations into strata of risk or burden of disease, and model outputs just for those target audiences. Consequently, health impact can be estimated for specific populations, including subgroups such as sex workers or most at-risk populations for HIV. The DALYs averted models are available upon request, including the coefficients, the item that partner organizations have typically preferred to request thus far. The majority of PSI's DALYs averted models are assembled and managed in a Microsoft Access model platform where products and services sharing the same benefits are grouped under the same model. Some remain only in Excel due to lacking of programming resources.

The results above demonstrate the utility of basing a program health impact measure on the DALY. Because burden of disease is incorporated into the calculation of DALYs averted, it is possible to compare program results from different countries as well as from different interventions. The most straightforward comparisons are those between countries offering the same health products and services. In these cases, the health impact of a product or service from one country is contrasted with the same product/service in another, with the varying disease burdens accounting for different impact results. As shown above, a water treatment intervention in Cameroon averts more cases, deaths, and DALYs attributable to diarrhea when compared with the same social marketing program in Cambodia due to Cameroon's higher under-five diarrhea mortality rate and its larger proportion of children under five in households.

Making health impact comparisons across different products/services with DALYs averted is more complicated, however. In these cases, the coefficients cannot be simply placed side by side because the product/service unit - and therefore, the length of coverage provided by the product/service - differs. One unit of the *PUR *water chlorination solution does not provide the same level of disease protection as that offered by one male condom. Moreover, if the definition of one unit of a product changes, such as from one condom to a pack of condoms, the DALYs averted coefficient changes accordingly. As a result, when comparing impact, simply looking at the magnitude of the coefficients without paying attention to the units can be misleading.

To overcome these challenges and make accurate and meaningful cross-product comparisons, the DALYs averted coefficients should be standardized against a common factor such as a population cohort or a cost unit. Cost per DALYs averted is a particularly effective measure as it allows programmers and decision makers to understand which intervention may yield the greatest impact at the lowest cost. For example, family planning programmers can use cross-intervention comparisons of cost per DALYs averted to assess the cost-effectiveness and relative health impact of distributing 10,000 male condoms through traditional outlets versus inserting 1,000 IUDs through health facilities. Once the product/service unit is properly considered and impact coefficients are standardized, a social marketing organization or a donor needing to identify program priorities based on maximum health impact can benefit from these types of comparisons.

Despite all of these advantages, the DALYs averted models are not without limitations. In their current form, these models are challenging to implement for several reasons, related to utility as well as the model parameters used. Currently, these models are intervention-specific, generating outputs for understanding the performance of a specific program. In doing so, the models achieve the purpose for which they were developed: to track program progress cumulatively within PSI contexts, specifically to estimate the impact of PSI programs among populations chosen by PSI. However, given the many players in disease prevention and control, such as implementing organizations and government agencies who also encourage the adoption of healthy behaviors, it is difficult to isolate PSI's impact in the models. While increased use of a PSI product may be the result of increased distribution or its accompanying BCC messages, this usage is also likely shaped by the efforts of other players. To address this problem in cases where health impact stems from interventions shared with other organizations, PSI employs its significant involvement policy to substantiate attribution and mitigate it (in terms of DALYs averted). Even so, these procedures probably do not capture all of the non-PSI activities that influence uptake of PSI products and services. Therefore, the health impact estimates produced by PSI's DALYs averted models may be slightly overstated in some cases.

Implementing organizations also must be able to account for the impact of multiple interventions on one health condition or for the benefits of a single intervention on multiple health conditions. By not doing so, they risk generating inaccurate assessments of program impact. Currently, only a few DALYs averted models incorporate cross-intervention synergy across disease areas, such as the basic care package for PLHIV model. In this model, the PLHIV can actually suffer from several diseases in addition to HIV infection, such as malaria and diarrhea. Therefore, the model considers the interaction between multiple diseases even though the person's risk of mortality is singular, i.e., a person can only die once even with concurrent diseases. Most of our models do not recognize this synergy, however, potentially resulting in overestimates of health impact. Similar problems affect the impact estimates for interventions in which the synergy crosses products and services. For example, the new infections averted that are claimed for both the condom and the HIV counseling and testing (HCT) interventions may be double counted due to HCT increasing the use of PSI condoms. Sometimes, this inattention to intervention synergy can lead to underestimates of health impact, if the model fails to acknowledge the indirect impacts of an intervention on other disease areas. For example, malaria prevention models predict the direct impact on malaria only; the models do not consider a bednet intervention's indirect impacts, such as the decline in anemia and intrauterine growth restriction associated with malaria disease burden reductions. Most models do not recognize these indirect impacts, instead emphasizing the health impact of the intervention on one condition only.

PSI's process for developing the models accounts for this current lack of synergy. With the exception of a few cases, most models were developed in isolation, product by product, service by service, and disease condition by disease condition. PSI's future program scope in terms of expansion into new disease areas was not considered. Compounding this challenge is the time needed to develop the models. PSI's DALYs averted models were developed at different time points in the past four to five years. In some cases, modeling occurred before the emergence of new evidence demonstrating the utility of an intervention across diseases (e.g., the efficacy of using male circumcision to reduce the transmission of STIs other than HIV) or the utility of multiple interventions on one condition (e.g., distributing condoms and providing and promoting HCT to reduce HIV transmission). As a result, the models limit health impact estimations to one disease only, not the actual impact that an intervention may have in a population. These current model drawbacks should be addressed in future years by ensuring the models incorporate synergy across interventions and disease areas, and by devising easier systems for model maintenance.

In terms of model inputs, the DALYs averted models are limited in several ways. First, the models are built on the best publicly available data at the time of development. For a given year, the models use published epidemiologic data of the diseases of interest and a common source of demographic data (usually UNPD figures). If these published data have not been updated or if modelers have not had the opportunity to apply the new evidence to the model parameters, the models remain static and may become outdated. For example, we developed the DALYs averted models on HIV prevention (including the HIV Condom Model described above) in 2007, when ART was not widely available in developing countries. Therefore, the models follow the standard disease progression of an individual not on ART, meaning we assume an infected person will live ten years with HIV infection and then two years of AIDS before dying. As this assumption no longer holds true, these models are likely overestimating the health impact of these HIV prevention interventions. While these DALYs averted models are in the process of being updated to account for ART coverage, they still rely on older data at the moment.

To address these issues of outdated evidence, it would be preferable to actively draw epidemiologic and demographic model inputs from a regularly updated database. At the moment, however, there are few publicly available, global health data sources offering estimates of disease incidence or prevalence at the country level. One option may be to use demographic and epidemiologic projections in the models themselves as these do not rely on outdated data. However, these projections have been out of scope for the PSI models to date and would be difficult to accommodate with the current models.

Relatedly, data are not always available to accommodate model parameters for all countries and study populations. The establishment of new countries, such as the recent establishment of South Sudan, can often leave the development community scrambling for viable data that estimate health or demographic parameters. In these cases, PSI can make assumptions or use proxies, although these may not accurately reflect the country or population of interest. For example, in the HIV Condom DALYs Averted Model focused on heterosexual transmission, we use a set of 'default' values obtained from pooled data of three PSI population-based surveys in Angola, Zimbabwe, and Zambia when sexual behavior data from the country of interest are not available. It is questionable whether this data based on an African population can be applied to other populations receiving HIV prevention programs, such as Asia and Latin America.

In addition to needing the most viable and up-to-date epidemiologic and demographic data, successful implementation of the models requires credible distribution data, for the final step of translating program outputs into program impacts. To avoid any inaccuracies in product distribution and service delivery numbers, proper internal program monitoring systems need to be established and adhered to, with reliable and complete data collected at all levels of the supply chain. As part of the reporting process, data managers need to use standard units for each product and service (e.g., one sachet of ORS instead of one box which may include multiple sachets). PSI is currently working on upgrading and standardizing its monitoring systems to make sure all country offices are reporting distribution data in the same way and in a consistent fashion. Implementing organizations that wish to adapt these DALYs averted models will benefit from considering the robustness and effectiveness of their monitoring systems in advance of using the models.

Many of these limitations can be addressed by incorporating output from external models into the DALYs averted models. The Lives Saved Tool (LiST) provides a model system that outputs deaths averted for nearly all of the PSI interventions aimed at children and maternal health. Tying estimates of DALYs averted to LiST ensures a common structure as well as a standard system for keeping models current. With population and epidemiological data already in its database, including the parameters associated with each disease condition, LiST's data are consolidated and regularly updated [9]. Therefore, adopting LiST would enable easy and routine updates to the DALYs averted model parameters and assumptions, reducing the reliance on limited staff for this work. LiST's structure also supports intervention synergy in its model system [9], so the DALYs averted models would reflect the dynamic environment they measure. Moreover, even though LiST's utility is limited to maternal and child health modeling needs [9], its robust and comprehensive database will simplify and speed up the modeling process for new interventions. PSI is currently exploring collaborations to standardize its DALYs averted currency against these models, developing a conversion model that would convert LiST outputs of lives saved to DALYs averted.

## Conclusion

PSI's DALYs averted models offer practical measurement solutions for implementing organizations in global health seeking to estimate the impact of their interventions. Developed as part of an ambitious program to demonstrate accountability in PSI's work, these models meet the unique needs of implementing organizations that previous intervention impact models had not successfully addressed: they predict the health impact of individual interventions covering a diverse range of health areas and they do so by directly estimating the impact of one product/service unit, data that implementing organizations often routinely track as program outputs. While improvements to the model inputs and modeling process are still needed, PSI's efforts thus far have demonstrated how DALYs averted can be viably used by the global health community as a metric for predicting and assessing health intervention impact using standard program output data. The model structure and approach established by PSI provides a standard, robust intervention impact modeling system based on the DALY. It also lays the groundwork for the development of new models that reflect the broadening and changing needs in global health, such as the expansion into non-communicable diseases that PSI and many other global health implementing organizations have recognized as essential new components of their intervention portfolios.

## List of abbreviations

PSI: Population Services International; DALYs: disability-adjusted life year; HIV: human immunodeficiency virus; TB: tuberculosis; BCC: behavior change communication; USAID: United States Agency for International Development; GBD: Global Burden of Disease; LiST: Lives Saved Tool; BOD: burden of disease; FP: family planning; AIDS: acquired immune deficiency syndrome; STI: sexually transmitted infection; HSV-2: herpes simplex virus-2; HPV: human papilloma virus; IDU: injection drug user; HBV: Hepatitis B virus; HCV: Hepatitis C virus; VIA: visual inspection with acetic acid; LBW/IUGR: low birthweight/intrauterine growth retardation; PAC: post-abortion care; IUD: intrauterine device; ARI: acute respiratory infection; ORS: oral rehydration solution; DTK: diarrhea treatment kit; HCT: HIV counseling and testing; PMTCT: prevention of mother-to-child transmission of HIV; ARV: antiretroviral; HAART: highly-active antiretroviral therapy; AZT: zidovudine; NVP: nevirapine; MC: male circumcision; PLHIV: people living with HIV; LLINs: long-lasting, insecticide-treated nets; ACT: artemisinin-based combination therapy; RDT: rapid diagnostic testing; DOTS: directly observed therapy, short-course; WHO: World Health Organization; DHS: Demographic and Health Survey; UNAIDS: Joint United Nations Programme on HIV/AIDS; CHERG: Child Health Epidemiology Reference Group; CYPs: couple-years of protection; HYP: household-years of protection; UNPD: United Nations Population Division; IMR: infant mortality rate; U5MR: under-five mortality rate; ART: antiretroviral therapy; FSW: female sex worker

## Competing interests

The authors declare that they have no competing interests.

## Authors' contributions

HY, WS, and DJ conceptualized and developed the models. HY, SD, and AR wrote the paper. HY produced the results. WS, DJ, AR, and SD reviewed and edited the paper. All authors read and approved the final manuscript.

## Supplementary Material

Additional file 1 **List of PSI DALYs averted models by intervention**. This table provides a comprehensive list of PSI's DALYs averted models, describing the interventions covered by the model, target populations, the health impact modeled, and the unit of product/service used to estimate impact. Click here for file

Additional file 2 **Adjustment calculations of HIV prevalence in commercial sex partners and the prevalence of STIs in the general adult population**. This file describes the adjustment calculations used to estimate HIV prevalence in commercial sex workers and the prevalence of STIs in the general adult population, for use in the HIV DALYs Averted Models. Click here for file

Additional file 3 **Calculating the per-act infectivity of HIV transmission**. This file describes the calculations used in the HIV Condom Model to calculate the per-act infectivity of HIV transmission, based on the various probabilistic combinations of the risk cofactors that an HIV-negative person may encounter in an HIV-positive partner (e.g., STI infection status in either partner, circumcision status of male partner). Click here for file

Additional file 4 **HIV DALYs averted for male condoms by all PSI programs implementing male condom interventions in 2012, by country**. This table shows the distribution data and DALYs averted by male condoms in each PSI country that implemented these interventions in 2012, based on the country-specific HIV DALYs averted coefficients for male condoms. Click here for file

Additional file 5 **Total DALYs averted by all PSI interventions worldwide, 2012, by intervention**. This table lists the 2012 global distribution figures of all PSI products/services and the total number of DALYs averted by each PSI intervention worldwide in 2012. Click here for file

## Declarations

This article is part of the supplement of *BMC Public Health *Volume 13, Supplement 2, 2013: Use of health impact metrics for programmatic decision making in global health. Population Services International, a registered non-profit organization, provided the funding for the publication of this supplement. The full contents of the supplement are available online at http://www.biomedcentral.com/bmcpublichealth/supplements/13/S2.

## References

[B1] Cole BL Fielding JE Health impact assessment: a tool to help policy makers understand health beyond health care Annu Rev Public Health 2007 28 393 412 10.1146/annurev.publhealth.28.083006.131942 17173539

[B2] The Global Fund: Performance Based Funding http://www.theglobalfund.org/en/activities/pbf/

[B3] US Agency for International Development: Performance-based Incentives Primer for USAID Missions http://pdf.usaid.gov/pdf_docs/PNADX747.pdf

[B4] The World Bank: Program-for-Results Financing http://web.worldbank.org/WBSITE/EXTERNAL/PROJECTS/0,,contentMDK:23215867~pagePK:41367~piPK:51533~theSitePK:40941,00.html

[B5] Murray CJ Ezzati M Flaxman AD Lim S Lozano R Michaud C Naghavi M Salomon JA GBD 2010: design, definitions, and metrics Lancet 2012 380 2063 2066 10.1016/S0140-6736(12)61899-6 23245602

[B6] UNAIDS/WHO/SACEMA Expert Group on Modelling the Impact and Cost of Male Circumcision for HIV Prevention Male circumcision for HIV prevention in high HIV prevalence settings: what can mathematical modelling contribute to informed decision making? PLoS Med 2009 6 9 10.1371/journal.pmed.1000109 PMC2731851 19901974

[B7] Correa H Beasley JD Mathematical models for decision-making in population and family planning Am J Public Health 1971 61 1 138 151 10.2105/AJPH.61.1.138 5539841 PMC1530640

[B8] Molineaux L Deitz K Thomas A Further epidemiological evaluation of a malaria model Bull World Health Organ 1978 56 4 565 71 365384 PMC2395644

[B9] Winfrey W McKinnon R Stover J Methods used in the Lives Saved Tool (LiST) BMC Public Health 2011 11 Suppl 3 S32 10.1186/1471-2458-11-S3-S32 21501451 PMC3231906

[B10] Jayakumar B Integrating maternal, newborn and child health interventions in Global Fund-supported programmes World Vision International 2011

[B11] The President's Emergency Plan for AIDS Relief PEPFAR Guidance on Integrating Prevention of Mother to Child Transmission of HIV, Maternal, Neonatal, and Child Health and Pediatric HIV Services 2011

[B12] Murray CJ Lopez AD Quantifying disability: data, methods and results Bull World Health Organ 1994 72 3 481 494 8062403 PMC2486704

[B13] Meekers D Van Rosen Explaining inconsistencies between data on condom use and condom sales BMC Health Serv Res 2005 5 5 10.1186/1472-6963-5-5 15651994 PMC545997

[B14] Myer L Mathews C Little F Tracing condom fates: design and pilot results of a study investigating the use and wastage of public sector condoms Afr J Reprod Health 2001 5 1 66 74 10.2307/3583199

[B15] Osterberg L Blaschke T Adherence to medication N Engl J Med 2005 353 487 497 10.1056/NEJMra050100 16079372

[B16] Walker N Fischer-Walker C Bryce J Bahl R Cousens S writing for the CHERG Review Groups on Intervention Effects Standards for CHERG reviews of intervention effects on child survival Int J Epidemiol 2010 39 i21 i31 10.1093/ije/dyq036 20348122 PMC2845875

[B17] Lopez AD Mathers CD Ezzati M Jamison DT Murray CJL Lopez AD, Mathers CD, Ezzati M, Jamison DT, Murray CJ Measuring the global burden of disease and risk factors, 1990-2001 Global Burden of Disease and Risk Factors 2006 Oxford University Press and The World Bank

[B18] Mathers CD Lopez AD Murray CJL Lopez AD, Mathers CD, Ezzati M, Jamison DT, Murray CJ The burden of disease and mortality by condition: data, methods, and results for 2001 Global Burden of Disease and Risk Factors 2006 Oxford University Press and The World Bank 21250373

[B19] Tumwine JK Thompson J Katua-Katua M Mujwajuzi M Johnstone N Porras I Diarrhoea and effects of different water sources, sanitation and hygiene behaviour in East Africa Trop Med Int Health 2002 7 9 750 6 10.1046/j.1365-3156.2002.00927.x 12225505

[B20] Thompson T Khan S Situation analysis and epidemiology of infectious disease transmission: a South-East Asian regional perspective Int J Environ Health Res 2003 13 Suppl 1 S29 36 12775377 10.1080/0960312031000102787

[B21] Clasen T Roberts I Rabie T Schmidt W Cairncross S Interventions to improve water quality for preventing infectious diarrhoea (a Cochrane Review) Cochrane Database Syst Rev 2006 3 10.1002/14651858.CD004794.pub2 16856059

[B22] PSI Research & Metrics Evaluating knowledge of HIV status among men and women in Zimbabwe TRaC Summary Report 2008

[B23] PSI Research & Metrics Examining the use of voluntary counseling and testing among women and men aged 15-49 in Zambia. Round one TRaC Summary Report 2007

[B24] Gray RH Wawer MJ Brookmeyer R Sewankambo NK Serwadda D Wabwire-Mangen F Lutalo T Li C vanCott T Quinn TC the Rakai Project Team Probability of HIV-1 transmission per coital act in monogamous, heterosexual, HIV-1-discordant couples in Rakai, Uganda Lancet 2001 357 1149 1153 10.1016/S0140-6736(00)04331-2 11323041

[B25] Satten GA Mastro TD Longini IM Modelling the female-to-male per-act HIV transmission probability in an emerging epidemic in Asia Stat Med 1994 13 2097 2016 10.1002/sim.4780131918 7846413

[B26] World Health Organization Mortality and Burden of Disease Estimates for WHO Member States in 2004 2009 WHO Department of Measurement and Health Information

[B27] Demographic and Health Surveys (DHS) http://www.measuredhs.com

[B28] Multiple Indicator Cluster Surveys (MICS) http://www.childinfo.org

[B29] UNAIDS Reports http://www.unaids.org/en/

[B30] World Health Organization Epidemiological Reports http://www.who.int/publications/en/

[B31] Population Services International TRaC Reports http://www.psi.org/trac

[B32] Child Health Epidemiology Reference Group Reviews http://cherg.org/publications.html

[B33] Cochrane Literature Reviews http://www.cochrane.org/cochrane-reviews

[B34] USAID CYP Conversion Factor for Family Planning Products http://transition.usaid.gov/our_work/global_health/pop/techareas/cyp.html

[B35] World Health Organization Global Health Observatory Data Repository 2011 http://apps.who.int/ghodata/?vid=720

[B36] United Nations Population Division World Population Prospects, the 2010 Revision 2011 New York http://esa.un.org/unpd/wpp/

[B37] World Health Organization World Health Statistics Web Site 2007 http://www.who.int/gho/publications/world_health_statistics/en/index.html

[B38] Black RE Cousens S Johnson HL Lawn JE Global, regional, and national causes of child mortality in 2008: a systematic analysis Lancet 2010 375 1969 87 10.1016/S0140-6736(10)60549-1 20466419

[B39] UNAIDS Global Report: UNAIDS Report on the Global AIDS Epidemic 2010. Annex 1: HIV and AIDS Estimates and Data 2009 and 2001 2010 UNAIDS http://www.unaids.org/GlobalReport/Global_report.htm

[B40] Williams BG Lloyd-Smith JO Gouws E Hankins C Getz WM Hargrove J de Zoysa I Dye C Auvert B The potential impact of male circumcision on HIV in sub-Saharan Africa PLoS Med 2006 3 7-e262 1032 40 10.1371/journal.pmed.0030262 PMC1489185 16822094

[B41] Boily MC Baggaley RF Wang L Masse B White RG Hayes RJ Alary M Heterosexual risk of HIV-1 infection per sexual act: systematic review and meta-analysis of observational studies Lancet Infect Dis 2009 9 118 129 10.1016/S1473-3099(09)70021-0 19179227 PMC4467783

[B42] Pilcher CD Brief but efficient: acute HIV infection and the sexual transmission of HIV J Infect Dis 2004 189 1785 92 10.1086/386333 15122514

[B43] Wawer MJ Gray RH Sewankambo NK Serwadda D Li X Laeyendecker O Kiwanuka N Kigozi G Kiddugavu M Lutalo T Nalugoda F Wabwire-Mangen F Meehan MP Quinn TC Rates of HIV-1 transmission per coital act, by stage of HIV-1 infection, in Rakai, Uganda J Infect Dis 2005 191 1403 1409 10.1086/429411 15809897

[B44] Rottingen JA Cameron DW Garnett GP A systematic review of the epidemiologic interactions between classic sexually transmitted diseases and HIV. How much really is known? Sex Transm Dis 2001 28 579 597 10.1097/00007435-200110000-00005 11689757

[B45] Pinkerton SD Abramson PR Effectiveness of condoms in preventing HIV transmission Soc Sci Med 1997 9 1303 1312 9141163 10.1016/s0277-9536(96)00258-4

[B46] Auvert B Taljaard D Lagarde E Sobngwi-Tambekou J Sitta R Puren A Randomized, controlled intervention trial of male circumcision for reduction of HIV infection risk: the ANRS 1265 trial PLoS Med 2005 11 1111 1122 10.1371/journal.pmed.0020298 PMC1262556 16231970

[B47] Bailey RC Moses S Parker CB Agot K Maclean L Krieger JN Williams CFM Campbell RT Ndinya-Achola JO Male circumcision for HIV prevention in young men in Kisumu, Kenya: a randomized controlled trial Lancet 2007 369 643 656 10.1016/S0140-6736(07)60312-2 17321310

[B48] Gray RH Kigozi G Serwadda D Makumbi F Watya S Nalugoda F Kiwanuka N Moulton LH Chaudhary MA Chen MZ Sewankambo NK Wabwire-Mangen F Bacon MC Williams CFM Opendi P Reynolds SJ Laeyendecker O Quinn TC Wawer MJ Male circumcision for HIV prevention in men in Rakai, Uganda: a randomised trial Lancet 2007 369 657 666 10.1016/S0140-6736(07)60313-4 17321311

[B49] Todd J Glynn JR Marston M Lutalo T Biraro S Mwita W Suriyanon V Rangsin R Nelson KE Sonnenberg P Fitzgerald D Karita E Zaba B Time from HIV seroconversion to death: a collaborative analysis of eight studies in six low and middle-income countries before highly active antiretroviral therapy AIDS 2007 21 suppl 6 S55 S63 10.1097/01.aids.0000299411.75269.e8 18032940 PMC5784803

