# Supplementary material for: Modeling health impact of global health programs implemented by Population Services International
Source: BMC Public Health. 2013 Jun 17;13(Suppl 2):S3. doi: 10.1186/1471-2458-13-S2-S3 (PMC3684543; doi:10.1186/1471-2458-13-S2-S3)
Supplement: Additional file 1 — List of PSI DALYs averted models by intervention. This table provides a comprehensive list of PSI's DALYs averted models, describing the interventions covered by the model, target populations, the health impact modeled, and the unit of product/service used to estimate impact. [file 1471-2458-13-S2-S3-S1.PDF]

Additional file 1. List of PSI DALYs averted models by intervention

Name of Model	Interventions Covered by Model	Target Populations	Health Impact Modeled	Product/Service Unit Used to Estimate Impact (DALYs averted coefficient = impact of one unit)
**Cervical Cancer**	Screening and referral	Women (15-49 yrs.)	Cervical cancer morbidity and mortality	Per screening and referral, by screening approach (i.e., visual inspection with acetic acid (VIA) or Pap smear)
	Screening and treatment	Women (15-49 yrs.)	Cervical cancer morbidity and mortality	Per screening and treatment, by screening approach (i.e., VIA or Pap smear) and abnormal status (less advanced or advanced)
**Maternal Nutrition**	Maternal iron folate	Pregnant women (15-44 yrs.)	1) Maternal morbidity and mortality associated with iron-deficiency anemia 2) Neonatal mortality associated with low birthweight/intrauterine growth retardation (LBW/IUGR)	Per tablet of iron folate
	Maternal multivitamin	Pregnant women (15-44 yrs.)	1) Maternal morbidity and mortality from eclampsia 2) Neonatal mortality associated with LBW/IUGR	Per multivitamin tablet
**Safe Delivery**	Clean Delivery Kit (CDK)	Pregnant women (15-44 yrs.)	Maternal morbidity and mortality from sepsis	Per CDK
	Misoprostol for post partum hemorrhage (PPH)	Pregnant women (15-44 yrs.)	Maternal morbidity and mortality from postpartum hemorrhage	Per pill
	Misoprostol for post abortion care (PAC)	Pregnant women (15-44 yrs.)	Maternal morbidity and mortaliity from abortion	Per pill
	Safe abortion (medical or surgical)	Pregnant women (15-44 yrs.)	All-cause maternal morbidity and mortality	Per abortion
**Family Planning**	Oral contraceptive	Women (15-44 yrs.)	1) Maternal morbidity and mortality 2) Under-five mortality associated with short birth intervals	Per menstrual cycle
	Contraceptive injection (1-,2-, and 3-month)	Women (15-44 yrs.)	Same as oral contraceptive	Per injection
	Male or female condom	Women (15-44 yrs.)	Same as oral contraceptive	Per condom

Name of Model	Interventions Covered by Model	Target Populations	Health Impact Modeled	**Product/Service Unit Used to Estimate Impact** (DALYs averted coefficient = impact of one unit)
**Family Planning**	Emergency contraception	Women (15-44 yrs.)	Same as oral contraceptive	Per dosage (2 tablets for one-time use)
	Voluntary surgical contraception	Women (15-44 yrs.)	Same as oral contraceptive	Per surgery
	Intrauterine device (IUD) (distribution or insertion)	Women (15-44 yrs.)	Same as oral contraceptive	Per IUD distributed or inserted
	CycleBeads	Women (15-44 yrs.)	Same as oral contraceptive	Per string of beads
	Hormone implant (distribution or insertion)	Women (15-44 yrs.)	Same as oral contraceptive	Per implant distributed or inserted
	Vaginal foaming tablet	Women (15-44 yrs.)	Same as oral contraceptive	Per tablet (discontinued product)
**Pneumonia Treatment**	Prepackaged antibiotics to manage acute respiratory infection (ARI)	The following with pneumonia symptoms:<br>Children under five<br>Children (5-14 yrs.)<br>Adults (≥ 15 yrs.)	Severe pneumonia morbidity and mortality	Per ARI package
**Micronutrient Supplement**	Micronutrients for treating nutritional anemia	Children under five	1) Anemia and iron-deficiency anemia morbidity<br>2) Diarrhea and ARI morbidity and mortality associated with iron-deficiency anemia	Per sachet
**Water Chlorination**	Water treatment with chlorination	Children under five<br>Children (5-14 yrs.)<br>Adults (≥ 15 yrs.)	Diarrhea morbidity and mortality	Per tab or bottle or sachet, depending on the product
**Diarrhea Treatment**	ORS (Oral rehydration solution)	Children under five with diarrhea symptoms	Diarrhea mortality	Per sachet
	Zinc	Children under five with diarrhea symptoms	Diarrhea mortality	Per pack of 7-10 tablets, depending on country-recommended dosage for one episode
	Diarrhea treatment kit (DTK) (2 sachets of ORS and 1 pack of zinc tablets)	Children under five with diarrhea symptoms	Diarrhea mortality	Per DTK
**HIV Condom**	Male or female condom	Adults (≥ 15 yrs.)	1) Heterosexual HIV transmission<br>2) HIV/AIDS morbidity and mortality	Per condom

Name of Model	Interventions Covered by Model	Target Populations	Health Impact Modeled	**Product/Service Unit Used to Estimate Impact** (DALYs averted coefficient = impact of one unit)
**HCT**	HIV counseling and testing (HCT)	Adults (≥ 15 yrs.)	1) Heterosexual HIV transmission<br>2) HIV/AIDS morbidity and mortality through behavior change<br>3) HIV/AIDS morbidity and mortality through ART treatment	Per HCT conducted, by HIV infection status and HCT uptake status (i.e., individual vs. couple)
**Male Circumcision**	Male circumcision	Adults (≥ 15 yrs.)<br>Neonates	1) Heterosexual HIV transmission<br>2) HIV/AIDS morbidity and mortality<br>3) HSV-2 morbidity<br>4) Morbidity and mortality from HPV-associated cancers	Per circumcision surgery provided, by HIV infection status
**STI Kit**	Sexually transmitted infection (STI) kits (includes antibiotics and condoms)	People with following STI syndromes: urethritis/cervicitis, non-herpetic genital ulcer, herpetic genital ulcer, vaginitis, lower abdominal pain, or inguinal bubo	1) STI morbidity and mortality<br>2) HIV heterosexual transmission<br>3) HIV/AIDS morbidity and mortality	Per kit
**PMTCT**	Three-stage prevention of mother-to-child transmission (PMTCT) of HIV (through antiretroviral (ARV) prophylaxis or therapy, elective Caesarean section, and replacement feeding)	HIV+ pregnant women	1) Mother-to-child HIV transmission (MTCT)<br>2) Heterosexual HIV transmission during pregnancy among sero-discordant couples for patients receiving highly-active retroviral therapy (HAART)<br>3) HIV/AIDS morbidity and mortality	Per delivery, by treatment administration status (i.e., mother-only versus mother and baby) and mother's ARV management status (i.e., zidovudine (AZT) prophylaxis, HAART, or single-dose nevirapine (NVP))
**HIV Behavior Change - ABC**	BCC focusing on Abstinence, Being faithful, and Condom use	Adults (≥ 15 yrs.) in general population and in most at-risk populations	1) HIV heterosexual transmission<br>2) HIV homosexual transmission (depending on study population)	Per intervention
**HIV Behavior Change - MC/HCT**	BCC to increase demand of MC or HCT	Adults (≥ 15 yrs.)	Same as MC and HCT, respectively	Per MC or HCT
**HAART**	HAART HIV treatment	Adults (≥ 15 yrs.) living with HIV/AIDS (PLHIV), with CD4 counts <350 and who are not pregnant	1) HIV heterosexual transmission<br>2) HIV morbidity and mortality	Per tablet of fixed-dose ARV combinations

Name of Model	Interventions Covered by Model	Target Populations	Health Impact Modeled	**Product/Service Unit Used to Estimate Impact** (DALYs averted coefficient = impact of one unit)
**Basic Care Package for PLHIV**	Basic care package for PLHIV (including male condoms, LLINs, household water treatment products, and oral rehydration salts)	Adult PLHIV	1) Heterosexual HIV transmission<br>2) HIV morbidity and mortality<br>3) Diarrhea morbidity and mortality among household members and PLHIV<br>4) Malaria morbidity and mortality among household members and PLHIV<br>5) Maternal morbidity and mortality<br>6) Under-five mortality due to birth spacing	Per package
**Needle/Syringe**	Needle/syringe distribution	Injection drug users (IDUs)	1) HIV/AIDS morbidity and mortality<br>2) Hepatitis B morbidity and mortality<br>3) Hepatitis C morbidity and mortality	Per needle/syringe
**Naloxone**	Naloxone emergency overdose treatment	IDUs	Opiate overdose mortality	Per ampoule of naloxone
**IDU Behavior Change - Initiation of Injection Drugs**	BCC to reduce initiation of injecting drug use	Drug users	1) HIV/AIDS morbidity and mortality<br>2) Hepatitis B morbidity and mortality<br>3) Hepatitis C morbidity and mortality	Per intervention
**IDU Behavior Change - Naloxone**	BCC to increase naloxone use upon overdose	IDUs	Opiate overdose mortality	Per intervention
**IDU Behavior Change - Needle/ syringe**	BCC to reduce sharing of needles/syringes	IDUs	1) HIV/AIDS morbidity and mortality;<br>2) Hepatitis B morbidity and mortality<br>3) Hepatitis C morbidity and mortality	Per intervention
**Malaria Prevention**	Long-lasting insecticide nets (LLINs)	Children under five<br>Children (5-14 yrs.)<br>Adults (≥ 15 yrs.), including pregnant women	1) Morbidity from malaria and severe malaria<br>2) Malaria mortality	Per net
**Antimalarial treatment**	Prepackaged Artemisinin-based combination therapy (ACT)	All people with fever among:<br>Children under five<br>Children (5-14 yrs.)<br>Adults (≥ 15 yrs.)	1) Morbidity from severe malaria<br>2) Malaria mortality	Per ACT package (one full-course treatment)

Name of Model	Interventions Covered by Model	Target Populations	Health Impact Modeled	**Product/Service Unit Used to Estimate Impact** (DALYs averted coefficient = impact of one unit)
	Prepackaged non-ACT antimalarial treatment	All people with fever among:<br>Children under five<br>Children (5-14 yrs.)<br>Adults (≥ 15 yrs.)	1) Morbidity from severe malaria<br>2) Malaria mortality	Per non-ACT package (one full-course treatment)
**Malaria Diagnosis**	Malaria rapid diagnostic test (RDT) kit	All people with fever among:<br>Children under five<br>Children (5-14 yrs.)<br>Adults (≥ 15 yrs.)	Malaria mortality (by increasing the probability that those treated with ACT or other antimalarial medicines are infected with malaria)	Per RDT kit
**Tuberculosis (TB)**	Directly Observed Treatment Short-course (DOTS)	Adults (≥ 15 yrs.)	TB morbidity and mortality	Per patient completing a full course of treatment
