# Supplementary material for: Modeling health impact of global health programs implemented by Population Services International
Source: BMC Public Health. 2013 Jun 17;13(Suppl 2):S3. doi: 10.1186/1471-2458-13-S2-S3 (PMC3684543; doi:10.1186/1471-2458-13-S2-S3)
Supplement: Additional file 2 — Adjustment calculations of HIV prevalence in commercial sex partners and the prevalence of STIs in the general adult population. This file describes the adjustment calculations used to estimate HIV prevalence in commercial sex workers and the prevalence of STIs in the general adult population, for use in the HIV DALYs Averted Models. [file 1471-2458-13-S2-S3-S2.PDF]

Additional file 2. Adjustment calculations of HIV prevalence in commercial sex partners and the prevalence of STIs in the general adult population

When data of HIV prevalence among commercial partners and the prevalence of STIs in the adult population and commercial sex partners are not readily available from published data sources, adjustment calculations are used.

Adjustment calculation for HIV prevalence in commercial sex partners

Different calculations are used to estimate HIV prevalence among commercial sex partners, depending on the region. First, we calculated the ratios of HIV prevalence in the general adult population to HIV prevalence among commercial sex partners for each of the countries that have data available. Then, we used these individual country ratios to simulate different equations for each WHO region. We chose the equation with the best fit for each region, using published literature to guide decision making when such data were available. For example, the equation for the sub-Saharan African region fit well with the data reported by Buve et al. (2001).

Below are the equations we derived for HIV prevalence among commercial sex partners for each region, where R_g is HIV prevalence in the adult population and R_c is HIV prevalence among female sex workers:

- Sub-Saharan African countries, $R_c = R_g \times (0.625 + \frac{35.987}{R_g \times 100})$;
- Asian countries, $R_c = R_g * 17$;
- Latin America and Caribbean countries, $R_c = R_g * (2.198 + 2.715 * R_g * 100)$

To derive these equations, we used data from the WHO/UNAIDS Epidemiological Fact Sheets on HIV/AIDS and STIs (2008).

Adjustment calculation for STI prevalence in general adult population

To develop the equation for determining STI prevalence in the general adult population, we used the following data: 1) HIV prevalence in the general population by region (UNAIDS/WHO, 2006) and 2) the STI prevalence rate for the general population by region (WHO, 2001). First, we calculated ratios of STI prevalence to HIV prevalence for all WHO regions. Using these ratios, we simulated different equations to identify the equation with the best fit. The one we chose is shown below:

$$S_g = R_g \times 4.6 \times (100 \times R_g)^{-0.382}$$

where R_g is HIV prevalence in the adult population and S_g is STI prevalence in the adult population.

For commercial sex partners, this adjustment calculation is not used. Instead, STI prevalence among commercial sex partners is set at 80% for PSI platforms lacking available data.
