# Supplementary material for: Modeling health impact of global health programs implemented by Population Services International
Source: BMC Public Health. 2013 Jun 17;13(Suppl 2):S3. doi: 10.1186/1471-2458-13-S2-S3 (PMC3684543; doi:10.1186/1471-2458-13-S2-S3)
Supplement: Additional file 3 — Calculating the per-act infectivity of HIV transmission. This file describes the calculations used in the HIV Condom Model to calculate the per-act infectivity of HIV transmission, based on the various probabilistic combinations of the risk cofactors that an HIV-negative person may encounter in an HIV-positive partner (e.g., STI infection status in either partner, circumcision status of male partner). [file 1471-2458-13-S2-S3-S3.PDF]

Additional file 3. Calculating the per-act infectivity of HIV transmission

A number of factors may influence the per-act infectivity of HIV transmission, including STI infection status within the partnership, HIV infection stage of the infected partner, circumcision status of the male partner, and condom usage during sexual intercourse. Per-act infectivity is therefore adjusted by considering the effects of such cofactors in various probabilistic combinations that an HIV-negative individual may encounter while having sex with an HIV-positive partner.

To adjust the per-act infectivity, we first calculate an intermediate probabilistic combination (i.e., $Semi_i$) of the risk cofactors other than condom usage during sexual intercourse. The following equation is used for this calculation:

$$\boldsymbol{Semi_i} = \left\{\left[\boldsymbol{p_{std\_partner_i}} \times \boldsymbol{\gamma_{no\_std}} \times \boldsymbol{E_{std}} + (\mathbf{1} - \boldsymbol{p_{std\_partner_{i)}}}) \times \boldsymbol{\gamma_{no\_std}}\right] \times \left[\mathbf{1} - \frac{\boldsymbol{D_{acute}}}{\mathbf{365} \times \boldsymbol{D_{HIVAIDS}}}\right] + \left[\boldsymbol{p_{std\_partner_i}} \times \boldsymbol{\gamma_{win\_nostd}} \times \boldsymbol{E_{std}} + \left(\mathbf{1} - \boldsymbol{p_{std\_partner_i}}\right) \times \boldsymbol{\gamma_{win\_nostd}}\right] \times \left[\frac{\boldsymbol{D_{acute}}}{\mathbf{365} \times \boldsymbol{D_{HIVAIDS}}}\right]\right\} * (1\text{-}\%MC * PE\ cir)$$

Where,

$p_{std\_partner}$: STI prevalence among partners

γ_{no_std}: Per-act transmission probability when neither partner has an STI

E_{std}: Effect of STI infection on HIV transmission probability, which equals 5

$p_{hiv\_partner}$: HIV prevalence among partners

D_{acute}: Duration of HIV acute infection stage, which is assumed to be 54 days

$D_{HIVAIDS}$: Duration of HIV/AIDS infection, which is assumed to be 12 years

γ_{win_nostd}: Per-act transmission probability when neither partner has STI, but the HIV infected partner is in acute stage of HIV infection

$\%MC$: Male circumcision rate

PE_{mc}: Protective efficacy of male circumcision

Then, γ_i is estimated as below:

$\gamma_i = semi_i \times (1 - PE_{cdm})$ when condom is used during sexual intercourse, or

$\gamma_i = semi_i$ when condom is not used during sexual intercourse.
