# Supplementary material for: Modeling health impact of global health programs implemented by Population Services International
Source: BMC Public Health. 2013 Jun 17;13(Suppl 2):S3. doi: 10.1186/1471-2458-13-S2-S3 (PMC3684543; doi:10.1186/1471-2458-13-S2-S3)
Supplement: Additional file 4 — HIV DALYs averted for male condoms by all PSI programs implementing male condom interventions in 2012, by country. This table shows the distribution data and DALYs averted by male condoms in each PSI country that implemented these interventions in 2012, based on the country-specific HIV DALYs averted coefficients for male condoms. [file 1471-2458-13-S2-S3-S4.PDF]

Additional file 4. HIV DALYs averted for male condoms by all PSI programs implementing a male condom intervention in 2012, by country

Country	HIV DALYs Averted Coefficients for Male Condoms	Male Condom Distribution, 2012	HIV DALYs Averted by Male Condoms, 2012
Angola	0.002793	7,722,752	21,567
Belize	0.002361	253,779	599
Benin	0.002315	9,474,758	21,930
Botswana	0.006008	3222809	19,362
Burundi	0.004268	2,434,410	10,391
C.A.R.	0.002927	4,494,528	13,156
Cambodia	0.000970	19,011,469	18,435
Cameroon	0.003683	21,427,386	78,923
Caribbean	0.000648	772,955	501
China	0.000210	262,482	55
Congo-Kinshasa	0.003894	61,476,182	239,415
Costa Rica	0.000127	1,092,387	138
Côte d'Ivoire (+AIMAS*)	0.003391	22,174,988	75,194
Dominican Republic	0.000555	13,427,624	7,456
El Salvador	0.000468	2,904,816	1,360
Guatemala	0.000468	10,211,611	4,781
Guinea	0.002319	9,318,180	21,612
Haiti	0.001742	2,124,288	3,700
Honduras	0.000468	3,680,152	1,723
India	0.000510	221,303,250	112,900
Kenya	0.002773	35,538,084	98,533
Laos	0.000197	4,918,704	971
Lesotho	0.005933	4,323,600	25,651
Liberia	0.002661	466,924	1,243
Madagascar	0.001962	9,070,108	17,799
Malawi	0.004954	6,238,990	30,909
Mali	0.002433	18,199,210	44,284
Mexico	0.000127	25,578	3
Mozambique	0.004998	18,244,756	91,187
Myanmar	0.001158	21,993,848	25,475
Nicaragua	0.000078	5,159,547	402
Nigeria	0.003018	213,739,536	645,122
Pakistan	0.000132	103,110,124	13,589
Panama	0.000555	3,201,440	1,778
Papua New Guinea	0.001822	942,383	1,717
Paraguay	0.000127	2,268,148	288
Romania	0.000956	4,335,875	4,145
Rwanda	0.004234	11,093,987	46,968
Senegal	0.002146	1,970,913	4,229
South Africa	0.004907	70,367,916	345,290
South Sudan	0.003243	1,288,580	4,179

Country	HIV DALYs Averted Coefficients for Male Condoms	Male Condom Distribution, 2012	HIV DALYs Averted by Male Condoms, 2012
Swaziland	0.006132	1,642,992	10,075
Tanzania	0.003862	68,724,288	265,423
Togo	0.002142	3,678,968	7,880
Uganda	0.004426	4,636,300	20,518
Viet Nam	0.000779	8,139,675	6,343
Zambia	0.005113	27,870,255	142,511
Zimbabwe	0.005238	27,925,290	146,264
**Total**		**1,095,906,825**	**2,655,973**

**AIMAS is l'Agence Ivorienne de Marketing Social, a local NGO in Côte d'Ivoire that PSI works with closely to implement male condom interventions.
