# Supplementary material for: Modeling health impact of global health programs implemented by Population Services International
Source: BMC Public Health. 2013 Jun 17;13(Suppl 2):S3. doi: 10.1186/1471-2458-13-S2-S3 (PMC3684543; doi:10.1186/1471-2458-13-S2-S3)
Supplement: Additional file 5 — Total DALYs averted by all PSI interventions worldwide, 2012, by intervention. This table lists the 2012 global distribution figures of all PSI products/services and the total number of DALYs averted by each PSI intervention worldwide in 2012. [file 1471-2458-13-S2-S3-S5.PDF]

Additional file 5. Total DALYs averted by all PSI interventions worldwide, 2012, by intervention

Intervention Products / Services	2012 Distribution	Total DALYs Averted* by Intervention in All PSI Countries, 2012
Long-lasting, insecticide-treated net	23,477,497	5,872,021
Condom	1,095,906,825	4,123,690^
Artemisinin-based combination therapy (malaria)	15,351,526	2,401,212
Free condom	196,885,149	848,278
Antibiotic treatment (pneumonia)	3,549,565	705,781
Oral contraceptives	47,642,305	461,619
Contraceptive injectable (3-month)	7,337,034	298,153
Male circumcision	185,813	228,677
Safe water solution (*Waterguard*)	15,080,483	196,637
IUD insertion (10-year)	315,168	184,821
Basic Care Package for HIV	11,762,090	166,932
HIV counseling and testing	1,735,376	160,510
Malaria rapid diagnostic tests	2,258,255	138,730
IUD distribution (5-year)	328,210	124,381
ORS and zinc treatment (diarrhea)	2,449,776	102,401
Contraceptive implant insertion (5-year)	159,157	84,917
IUD distribution (5-year)	139,323	80,407
TB DOTS	19,921	64,972
IUD insertion (5-year)	125,034	56,604
Contraceptive injectable (2-month)	1,732,173	53,931
Medication abortion	434,627	49,721
Female condoms	8,018,070	43,494
SDM (Cycle Beads)	124,982	41,334
Oral rehydration solution (ORS for diarrhea)	2,547,666	37,350
STI Kit	481,679	22,653
Water treatment tablets (*Aquatab*)	69,663,648	22,059
Emergency contraception	2,943,169	17,508
Zinc (diarrhea)	2,177,983	16,012
Contraceptive referral (implant and IUD)**	78,174	15,215
Retreatment of ITNs with *IconMaxx*	700,065	10,323
Implant insertion (3-month)	27,281	9,905
Sprinkles (micronutrient powder)	348,285	9,717
Misoprostol for postpartum hemorrhage	343,762	9,060
Voluntary surgical contraception	7,099	8,638
Contraceptive implant distribution (4-year)	15,639	8,236
Clean needle and syringe	1,800,950	6,928
Chloroquine (malaria in Haiti)	181,700	6,714
Contraceptive implant distribution (5-year)	22,434	6,180
Behavior change (HIV)^	n/a	6,082
Water purification with *PUR*	44,346,677	5,954
Naloxone (opiate overdose)	7,019	5,902
Free female condom	937,466	5,389
Contraceptive implant distribution (3-year)	7,474	4,953

Intervention Products / Services	2012 Distribution	Total DALYs Averted* by Intervention in All PSI Countries, 2012
Retreatment of ITNs with KO123	63,104	3,898
Contraceptive injectable (1-month)	274,227	3,244
Iron folic acid tablet	1,946,610	1,060
Clean delivery kit	73,306	865
Early infant male circumcision	546	571
Misoprostol for post-abortion care	310,408	289
Multivitamins through antenatal clinics	2,460,270	254
Manual vacuum aspiration	644	144
Antiretroviral treatment	52,380	131
Retreatment of ITNs (6-month)	5,000	53
Cervical cancer VIA and cryotherapy	8	0.8
**Total**	**36,091,496**	**16,734,511**

* DALYs are calculated based on the methodology used for the 1990 Global Burden of Disease (i.e., DALY (0.03,0)). It is not calculated based on the new GBD methodology that was released in late 2012.

** DALYs averted listed here include those averted from both HIV and family planning.

^ Distribution data are not available for BCC interventions. Instead, we use data from BCC interventions shown to be effective in effecting behavior change. In 2012, there were eight BCC interventions that demonstrated effectiveness in changing sexual behaviors (i.e., Abstinence, Being faithful, or Condom use with different type of partner) within the study population. We input data from these interventions into the HIV BCC model to estimate the number of DALYs averted by the behavior change activities.
